# Trends in STI testing and diagnosis rates during the COVID-19 pandemic at a large urban tertiary care center, and the role of the emergency department in STI care

**DOI:** 10.3389/frph.2023.1082429

**Published:** 2023-02-20

**Authors:** Kimberly A. Stanford, Joseph A. Mason, Eleanor E. Friedman

**Affiliations:** ^1^ Department of Medicine, Section of Emergency Medicine, University of Chicago, Chicago, IL, United States; ^2^ Department of Medicine, Section of Infectious Diseases and Global Health, University of Chicago, Chicago, IL, United States

**Keywords:** sexually transmitted infections, emergency department (ED), gonorrhea, chlamydia, trichomonas, pregnant women, COVID- 19

## Abstract

**Introduction:**

The COVID-19 pandemic has had profound effects on access to care, including outpatient sexually transmitted infection (STI) testing and treatment. Many vulnerable populations already relied on the emergency department (ED) for much of their care prior to the pandemic. This study examines trends in STI testing and positivity before and during the pandemic at a large urban medical center and evaluates the role of the ED in providing STI care.

**Methods:**

This is a retrospective review of all gonorrhea, chlamydia, and trichomonas tests from November 1, 2018, through July 31, 2021. Demographic information and location and results of STI testing were extracted from the electronic medical record. Trends in STI testing and positivity were examined for 16 month periods before and after the COVID-19 pandemic started (March 15, 2020), with the latter divided into the early pandemic period (EPP: March 15 -July 31, 2020) and late pandemic period (LPP: August 1, 2020 - July 31, 2021).

**Results:**

Tests per month decreased by 42.4% during the EPP, but rebounded by July 2020. During the EPP, the proportion of all STI testing originating in the ED increased from 21.4% pre-pandemic to 29.3%, and among pregnant women from 45.2% to 51.5%. Overall STI positivity rate increased from 4.4% pre-pandemic to 6.2% in the EPP. Parallel trends were observed for gonorrhea and chlamydia individually. The ED represented 50.5% of overall positive tests, and as much as 63.1% of positive testing during the EPP. The ED was the source of 73.4% of positive tests among pregnant women, which increased to 82.1% during the EPP.

**Conclusions:**

STI trends from this large urban medical center paralleled national trends, with an early decrease in positive cases followed by a rebound by the end of May 2020. The ED represented an important source of testing for all patients, and especially for pregnant patients, throughout the study period, but even more so early in the pandemic. This suggests that more resources should be directed towards STI testing, education, and prevention in the ED, as well as to support linkage to outpatient primary and obstetric care during the ED visit.

## Introduction

The COVID-19 pandemic has had profound effects on access to health care, including testing and treatment for sexually transmitted infections (STIs), the long-term implications of which are still unknown. STI care was especially affected early in the pandemic, when many outpatient clinics closed, stay-at-home orders were put in place, and there was widespread fear of accessing medical facilities. This may have led to delays in seeking care, difficulty accessing lab testing or medical treatment for STIs (1), or preferential utilization of the emergency department (ED) for STI care, as EDs remained open for in-person care throughout the pandemic. Even before the COVID-19 pandemic, the ED was already the preferred location of care for many vulnerable populations. Residents in areas of high economic hardship may disproportionately visit the ED for all health care (2), and Medicaid beneficiaries have been found to utilize the ED at twice the rate of those with private insurance (3), believing ED care to be more accessible, more affordable, and higher quality (4). Despite this preference, previous studies have suggested that the minority of STI care is provided through the ED (5–7).

This study took place at a large, urban, tertiary care center that includes inpatient and surgical facilities, an emergency department, and many affiliated outpatient clinics, which together care for more than 600,000 patients per year, including around 100,000 annual ED encounters. The hospital serves patients from neighborhoods with extreme economic hardship. The ED has had a universal HIV and syphilis screening program in place since 2019. It was previously reported that rates of both acute HIV (8) and syphilis (9) diagnosed through the ED's routine screening program increased early in the COVID-19 pandemic. However, gonorrhea, chlamydia, and trichomonas are still diagnosed through targeted testing at clinician discretion, and it is unknown if rates would increase in parallel with HIV and syphilis. National data from the Centers for Disease Control and Prevention (10) showed a dip in gonorrhea and syphilis cases from the beginning of the pandemic until the end of May 2020 compared to the same time period in 2019, followed by a sustained increase as compared to the previous year. National data indicates that chlamydia cases remained lower than previous throughout the year, although this is thought to represent decreased testing rather than lower infection rates.

Given that this study site serves a socioeconomically vulnerable population largely comprised of racial and ethnic minorities, there may be a greater likelihood that patients in this community disproportionately utilize the ED for STI care. This reliance on the ED may have continued or even increased during the COVID-19 pandemic. Unique cultural and economic pressures in this population might also lead to differing temporal trends in STIs, with cases increasing more quickly than the national trend, similar to previous findings for HIV and syphilis. This could have profound impacts on resource allocation, suggesting that EDs located in vulnerable communities should be provided with additional resources for STI testing, education, and prevention.

## Methods

Retrospective electronic medical record (EMR) data was obtained by selecting all encounters with a gonorrhea, chlamydia, or trichomonas test ordered between November 1, 2018, and July 31, 2021, for patients 18 years and older. Tests originated from all hospital locations, including outpatient primary care and specialty clinics, inpatient wards, and the ED, as well as satellite locations affiliated with the main hospital center. In this hospital system, the orders for gonorrhea and chlamydia nucleic acid amplification tests (NAAT), the primary means of testing for these infections, are linked, and one cannot be ordered without the other, but trichomonas is ordered separately on the same sample. All tests were ordered as part of routine clinical care at clinician discretion. There were no specific protocols in place to guide testing, with the exception of a temporary recommendation to limit testing during a national shortage of NAAT kits from September 2020 to January 2021. In response to this, hospital guidelines were created to treat symptomatic male patients and asymptomatic patients with reported STI exposures empirically without confirmatory testing. The COVID-19 pandemic was defined as beginning on March 15, 2020, as this was the date of the first local case and beginning of stay-at-home orders in the Chicago area. Trends in STI testing and positivity rates were examined for the 16.5-month periods before and after the pandemic started, with the latter divided into the early pandemic period (EPP), defined as March 15 through July 31, 2020, and the late pandemic period (LPP), from August 1, 2020, to July 31, 2021.

### Outcome measures

The primary outcomes for this analysis were STI testing trends (defined as number of tests per month) and STI positivity rates (defined as number of positives per test resulted).

### Covariates

Demographic information, including age, legal sex, race/ethnicity, as well as the location and results of STI tests were extracted from the EMR. The EMR did not record information about gender identity during the study period. Due to the nature of data extraction from the EMR, information about individual patient symptoms or chief complaint was not available. Pregnancy was defined as a positive urine pregnancy test ordered within 1 week prior to or after an STI test order. Due to the nature of EMR data extraction, pregnancy status by patient report, ultrasound, positive testing during an encounter earlier in pregnancy, or by serum pregnancy testing was not available, nor was the gestational age of pregnant patients. Young patients were defined as those between 18 and 35 years old.

### Statistical analysis

This analysis compared the distribution of patient characteristics and STI testing and positivity rates between the pre-pandemic period, EPP and LPP. Prevalence ratios were created using modified Poisson regression (11) and are reported for the EPP as compared to the pre-pandemic period. Data were analyzed by encounter rather than by individual patients, as some patients had multiple encounters during the study period. Data analysis was performed using R version 4.2.1.

This study was approved by the University of Chicago Institutional Review Board. This project was supported in part by the National Center for Advancing Translational Sciences (NCATS) of the National Institutes of Health (NIH) through Grant Number 5UL1TR002389–04 that funds the Institute for Translational Medicine (ITM).

## Results

**Figure 1:**
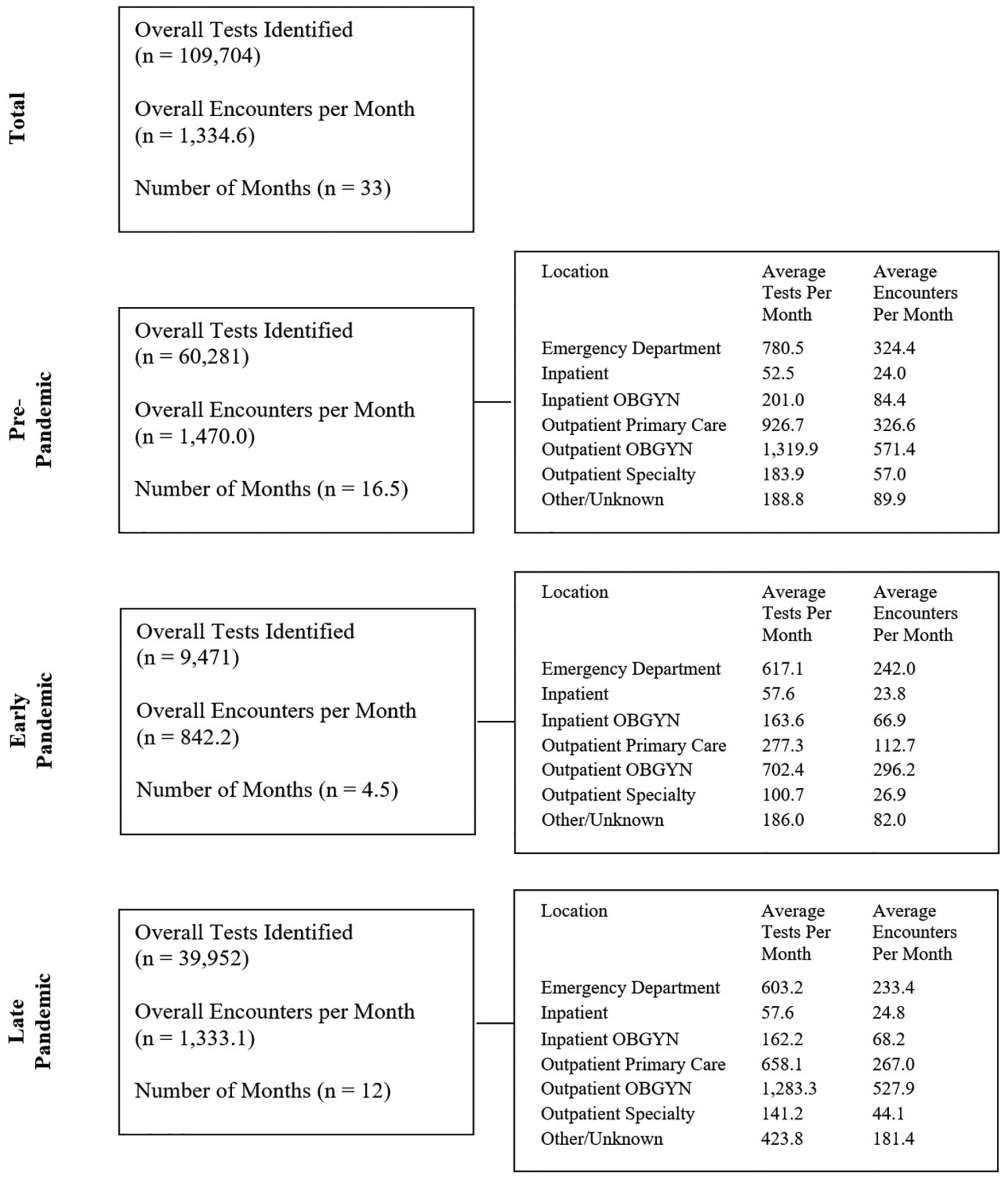
Number of STI tests and encounters for STI testing during the pre-pandemic, early pandemic and late pandemic periods, and rates of STI encounters and STI tests for each period by location of testing.

During the 33-month study period, 44,042 encounters for 29,880 unique patients were identified, with a median of 1 (IQR: 1–2) repeat encounters and 3 (IQR: 2–4) STI tests per patient. During these encounters, a total of 109,704 STI tests were performed, including 47,531 chlamydia tests, 47,533 gonorrhea tests, and 14,640 trichomonas tests. Figure 1 illustrates the testing and encounter rates by location and time period. The demographic makeup of overall patient encounters was predominantly non-Hispanic Black (NHB) (58.77%, 25,884/44,042) and female (77.03%, 33,926/44,042) with a median age of 28 years old (IQR: 23–35).

### Testing rates

**Figure 2:**
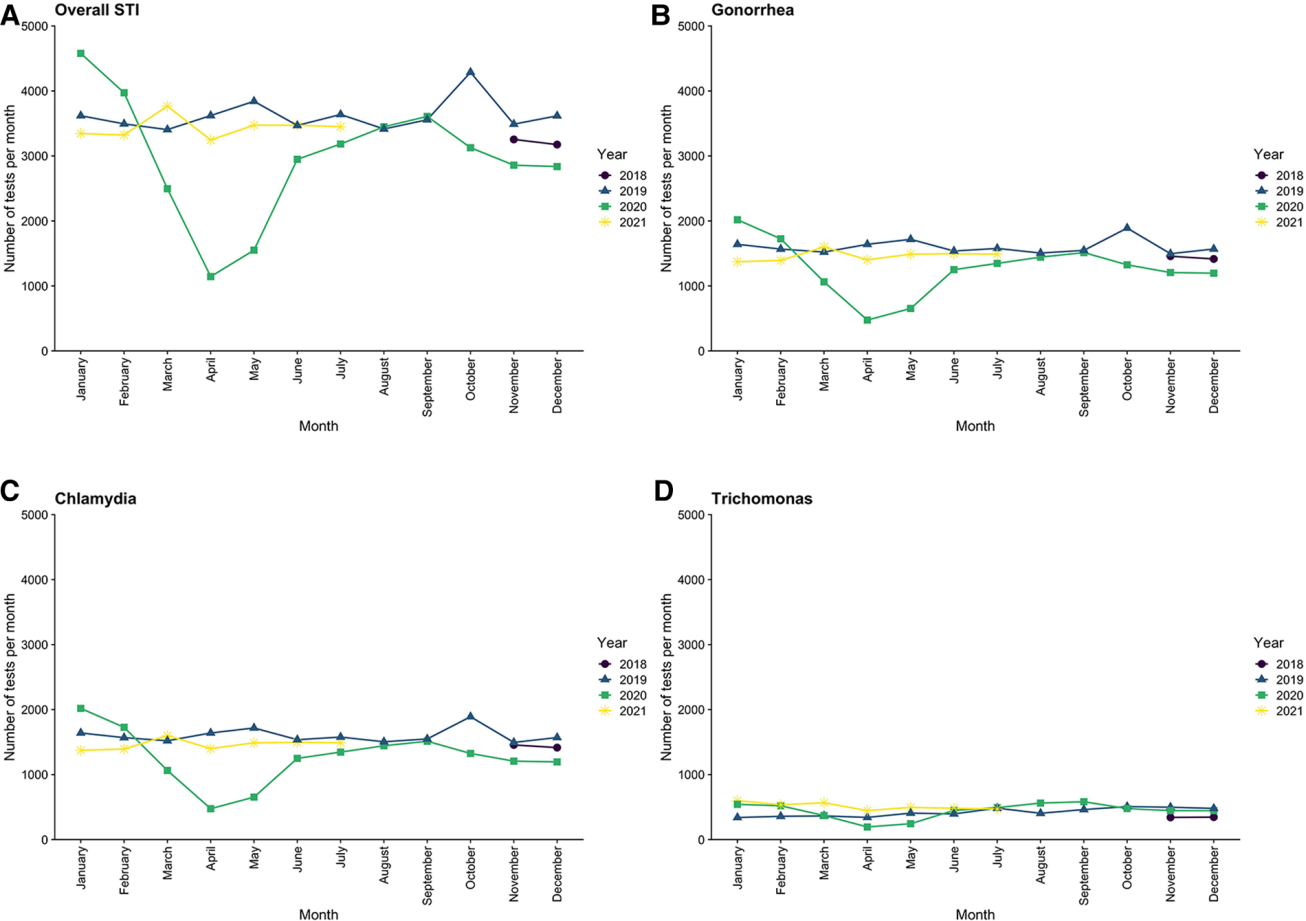
Trends in testing rates for sexually transmitted infections across the entire medical center by month, from November 2018 through July 2021.

The overall rate of STI testing decreased from a baseline of 3,653 tests per month pre-pandemic to 2,105 tests per month during the EPP (a decrease of 42.4%) but rebounded to pre-pandemic levels by the end of July 2020, with an average of 3,329 tests per month during the LPP (Figure 2). This pattern was seen individually for all three types of STI, with gonorrhea and chlamydia each decreasing by 45.0%, while trichomonas only decreased 22.6% during the EPP. All rebounded to near pre-pandemic levels in the LPP. The distribution of age, gender, and ethnicity of patients tested for STIs in the entire study population (Table 1) was similar between the pre-pandemic period and the LPP. However, during the EPP, there was an increase in the proportion of NHB (67.4% EPP vs. 56.4% pre-pandemic, PR 1.55, CI: 1.47–1.63, *p* < 0.001) and female (77.2% EPP vs. 73.7% pre-pandemic, PR 1.18, CI: 1.13–1.23, *p* < 0.001) patients tested for STIs (Table 2).

**Table 1:** Demographic characteristics of patients tested for gonorrhea, chlamydia, or trichomonas from November 1, 2018, through July 31, 2021, by COVID-19 time period: pre-pandemic (November 1, 2018 - March 14, 2020), early pandemic (March 15 - July 31, 2020), and late pandemic (August 1, 2020 - July 31, 2021).

Variable	Total Tests (*N*, %) *N* = 109,704	Tests by Time Period (*N*, %)		
		Pre-Pandemic *N* = 60,281	Early Pandemic *N* = 9,471	Late Pandemic *N* = 39,952
Gender				
Male	28,144 (25.65%)	15,865 (26.32%)	2,163 (22.84%)	10,116 (25.32%)
Female	81,551 (74.34%)	44,416 (73.68%)	7,308 (77.16%)	29,827 (74.66%)
Unknown	9 (0.01%)	0 (0%)	0 (0%)	9 (0.02%)
Race/Ethnicity				
Non-Hispanic Black	64,599 (58.88%)	33,997 (56.40%)	6,387 (67.44%)	24,215 (60.61%)
Non-Hispanic White	23,297 (21.24%)	14,101 (23.39%)	1,602 (16.91%)	7,594 (19.01%)
Non-Hispanic Other	13,751 (12.53%)	7,693 (12.76%)	838 (8.85%)	5,220 (13.07%)
Hispanic Black	418 (0.38%)	254 (0.42%)	46 (0.49%)	118 (0.30%)
Hispanic White	3,581 (3.26%)	2,058 (3.41%)	252 (2.66%)	1,271 (3.18%)
Hispanic Other	4,058 (3.70%)	2,178 (3.61%)	346 (3.65%)	1,534 (3.84%)
Age				
18 to < 20	7,975 (7.27%)	4,764 (7.90%)	721 (7.61%)	2,490 (6.23%)
20 to < 25	27,236 (24.83%)	15,730 (26.09%)	2,213 (23.37%)	9,293 (23.26%)
25 to < 30	27,616 (25.17%)	15,255 (25.31%)	2,441 (25.77%)	9,920 (24.83%)
30 to < 35	18,207 (16.60%)	9,291 (15.41%)	1,644 (17.36%)	7,272 (18.20%)
35 to < 45	17,702 (16.14%)	9,183 (15.23%)	1,605 (16.95%)	6,914 (17.31%)
45 to < 55	6,812 (6.21%)	3,812 (6.32%)	518 (5.47%)	2,482 (6.21%)
55 to < 65	3,034 (2.77%)	1,683 (2.79%)	228 (2.41%)	1,123 (2.81%)
>= 65	1,122 (1.02%)	563 (0.93%)	101 (1.07%)	458 (1.15%)
Pregnant During Testing				
Pregnant	7,041 (6.42%)	3,802 (6.31%)	734 (7.75%)	2,505 (6.27%)
Not Pregnant	102,663 (93.58%)	56,479 (93.69%)	8,737 (92.25%)	37,447 (93.73%)
Location				
Emergency Department	22,893 (20.87%)	12,878 (21.36%)	2,777 (29.32%)	7,238 (18.12%)
Inpatient	1,817 (1.66%)	867 (1.44%)	259 (2.73%)	691 (1.73%)
Inpatient OBGYN	5,999 (5.47%)	3,317 (5.50%)	736 (7.77%)	1,946 (4.87%)
Outpatient Primary Care	24,436 (22.27%)	15,291 (25.37%)	1,248 (13.18%)	7,897 (19.77%)
Outpatient OBGYN	40,339 (36.77%)	21,778 (36.13%)	3,161 (33.38%)	15,400 (38.55%)
Outpatient Specialty	5,182 (4.72%)	3,035 (5.03%)	453 (4.78%)	1,694 (4.24%)
Other/Unknown	9,038 (8.24%)	3,115 (5.17%)	837 (8.84%)	5,086 (12.73%)
Insurance				
Medicare/Medicaid	31,913 (29.09%)	16,137 (26.77%)	3,600 (38.01%)	12,176 (30.48%)
Private	63,809 (58.16%)	34,544 (57.30%)	5,068 (53.51%)	24,197 (60.57%)
Self-Pay	10,645 (9.70%)	7,592 (12.59%)	582 (6.15%)	2,471 (6.18%)
Unknown	3,337 (3.04%)	2,008 (3.33%)	221 (2.33%)	1,108 (2.77%)

**Table 2:** Prevalence ratios for STI testing and positivity rates for the early pandemic period compared to the pre-pandemic period.

	Testing Rates			Positivity Rates		
	PR	95% CI	*P*-value	PR	95% CI	*P*-value
Race/Ethnicity						
Non-Hispanic White	Ref			Ref		
Non-Hispanic Black	1.55	1.47–1.63	< 0.001	1.94	1.27–2.97	< 0.01
Other	1.06	0.99–1.14	0.07	0.66	0.37–1.17	0.16
Gender						
Male	Ref			Ref		
Female	1.18	1.13–1.23	< 0.001	0.87	0.74–1.01	0.07
Age						
Patients over age 35	Ref			Ref		
Young patients (< 35)	0.97	0.93–1.02	0.21	1.08	0.87–1.35	0.48
Insurance						
Medicaid/Medicare	Ref			Ref		
Private	0.70	0.67–0.73	< 0.001	0.64	0.53–0.76	< 0.001
Self-Pay/Missing	0.42	0.39–0.46	< 0.001	0.58	0.46–0.72	< 0.001
*Testing and Positivity by Location of Testing*						
All tests						
All others	Ref			Ref		
Emergency Department	1.43	1.38–1.49	< 0.001	1.57	1.35–1.83	< 0.001
Women						
All others	Ref			Ref		
Emergency Department	1.27	1.21–1.33	< 0.001	1.46	1.21–1.76	< 0.001
Pregnant women						
All others	Ref			Ref		
Emergency Department	1.24	1.08–1.41	< 0.01	1.36	0.64–2.89	0.43
Young patients						
All others	Ref			Ref		
Emergency Department	1.44	1.38–1.51	< 0.001	1.55	1.31–1.82	< 0.001
Non-Hispanic Black						
All others	Ref			Ref		
Emergency Department	1.22	1.16–1.27	< 0.001	1.36	1.15–1.60	< 0.001

Overall, 20.9% of tests were ordered in the ED. During the EPP, the proportion of STI testing originating in the ED increased from 21.4% pre-pandemic to 29.3% (vs. pre-pandemic, PR 1.43, CI: 1.38–1.49, *p* < 0.001), then decreased to 18.1% in the LPP, slightly below pre-pandemic levels. A similar trend was seen in all subgroups when analyzed by race/ethnicity, gender, and age.

For women, 20.7% of all STI tests were ordered from the ED. The proportion of STI tests for women originating in the ED increased from 22.0% pre-pandemic to 27.2% in the EPP (PR 1.27, CI: 1.21–1.33, *p* < 0.001), then decreased to 17.1% in the LPP. Notably, 41.1% of all STI tests for pregnant women originated in the ED during the observed period. The proportion of STI tests for pregnant women performed in the ED increased from 45.2% pre-pandemic to 51.5% during the EPP (PR 1.24, CI: 1.08–1.41, *p* < 0.01), before decreasing to 31.7% in the LPP, with almost the entirety of the remainder originating from outpatient obstetrics clinics during all periods. Demographics of pregnant women tested for STIs were slightly different from the population as a whole, as pregnant women were more often NHB (69.9% vs. 58.1%), more frequently utilized Medicare/Medicaid (38.7% vs. 28.4%), and almost no pregnant women were over the age of 45.

### Positivity rates

**Figure 3:**
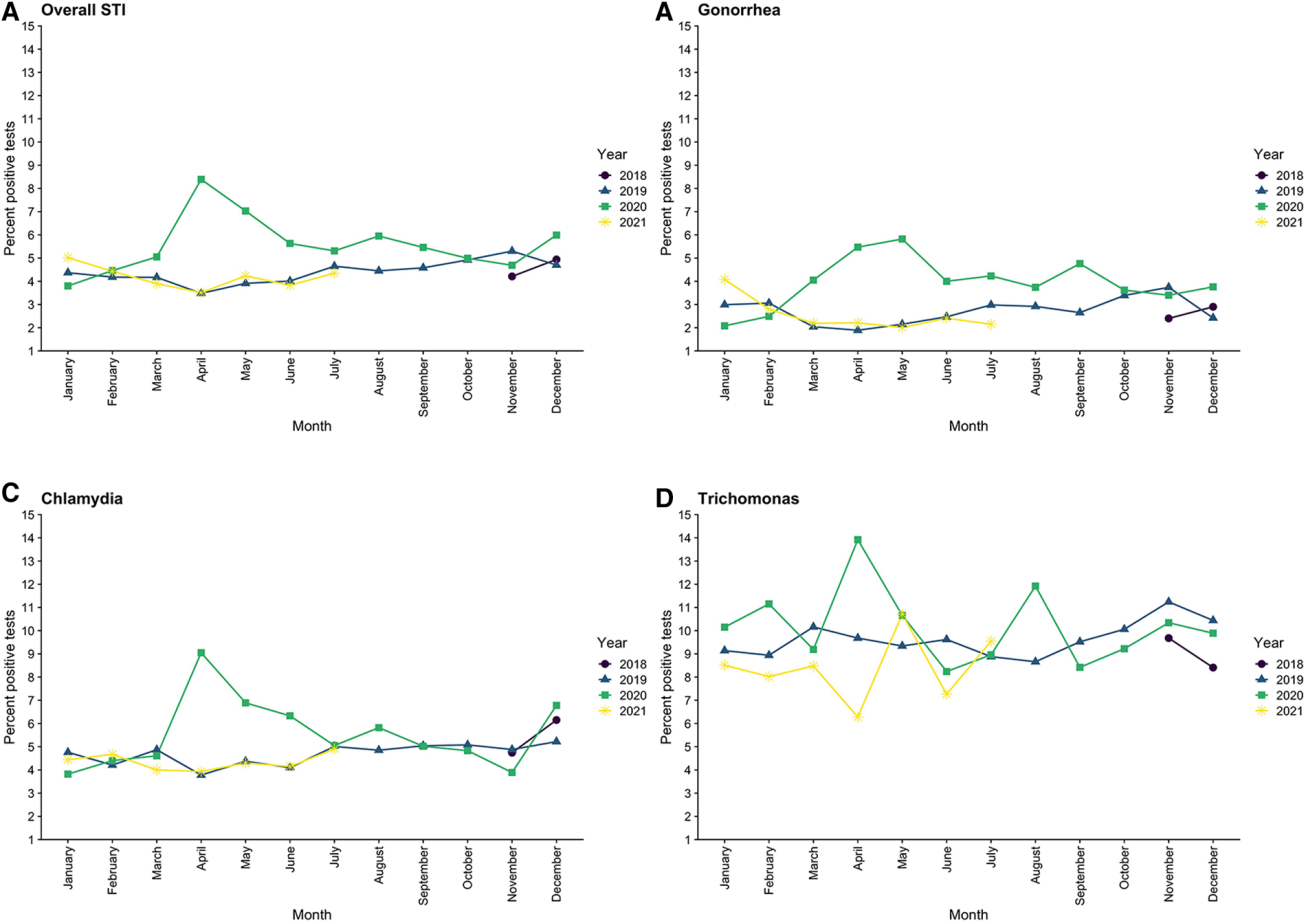
Trends in positivity rates for sexually transmitted infections across the entire medical center by month, from November 2018 through July 2021.

There was a large increase in positivity rate of all STI tests, which rose from 4.4% pre-pandemic to 6.2% in the EPP, followed by a decrease to 4.7% in the LPP, which was still above pre-pandemic baseline (Table 3). Parallel trends were observed for gonorrhea (positivity rate increased from 2.7% pre-pandemic to 4.6% in the EPP) and chlamydia (increased from 4.7% pre-pandemic to 6.3% in the EPP) individually, both of which returned to near baseline in the LPP (Figure 3). Trichomonas positivity rate, however, stayed fairly constant throughout (9.7% pre-pandemic to 9.8% EPP to 9.1% LPP). Over the entire study period, the distribution of age and gender of patients who tested positive for STIs was similar, however during the EPP, NHB patients represented 92.7% of positive tests, compared to 81.6% pre-pandemic (PR 1.94, CI: 1.27–2.976, *p* < 0.01) (Table 2).

**Table 3:** Outcomes of STI testing of patients tested for gonorrhea, chlamydia, or trichomonas from November 1, 2018, through July 31, 2021, by COVID-19 time period.

	Total Tests (*N*, %) *N* = 109,704	Tests by Time Period (*N*, %)		
		Pre-Pandemic *N* = 60,281	Early Pandemic *N* = 9,471	Late Pandemic *N* = 39,952
All STI Tests				
Positive	5,090 (4.64%)	2,637 (4.37%)	585 (6.18%)	1,868 (4.68%)
Negative	104,614 (95.36%)	57,644 (95.63%)	8,886 (6.18%)	38,084 (95.32%)
Gonorrhea Tests				
Positive	1,418 (2.98%)	714 (2.68%)	185 (4.63%)	519 (3.07%)
Negative	46,115 (97.02%)	25,902 (97.32%)	3,808 (95.37%)	16,405 (96.93%)
Chlamydia Tests				
Positive	2,288 (4.81%)	1,238 (4.65%)	254 (6.36%)	796 (4.70%)
Negative	45,243 (95.19%)	25,380 (95.35%)	3,737 (93.64%)	16,126 (95.30%)
Trichomonas Tests				
Positive	1,384 (9.45%)	685 (9.72%)	146 (9.82%)	553 (9.06%)
Negative	13,256 (90.55%)	6,362 (90.28%)	1,341 (90.18%)	5,553 (90.94%)

To assess if the increase in positivity rate simply reflected the decrease in testing volume during the EPP, the absolute testing numbers during the months of the EPP were compared to the same months in the years before and after. During the two first months of the pandemic, April and May 2020, the average absolute number of positive STI tests per month dropped precipitously to 102.5 positive tests per month, compared to 138 positives per month in 2019, and 130.5 in the same months in 2021. However, from June through August of 2020 the average absolute number of positives was much higher than the previous and following years (180.0 positives per month in 2020, compared to 153.3 in 2019, and 141.5 in June and July 2021 (data was not available for August 2021). This trend was also seen in subgroup analyses for women and within each age category.

The ED was an important source of positive testing, representing 50.5% of overall positive tests, and as much as 63.1% of positive testing during the EPP (PR 1.57, CI 1.35–1.83, *p* < 0.001). The next most frequent source of positive testing was outpatient obstetrics and gynecology clinic, which was the source of 18.4% of all positive tests. Importantly, the ED was the source of 73.4% of positive STI tests among pregnant women pre-pandemic, which increased to 82.1% during the EPP, however this change was not significant (PR 1.36, CI: 0.64–2.89, *p* = 0.43). During the entire study period, 243 STIs were diagnosed among 205 unique pregnant women visiting the ED. The trend towards an increase in the proportion of positive tests originating in the ED during the EPP held true in subgroup analyses for all women (PR 1.46, CI: 1.21–1.76, *p* < 0.001), young patients (PR 1.55, CI: 1.31–1.82, *p* < 0.001), and NHB patients (PR 1.36, CI: 1.15–1.60, *p* < 0.001).

## Discussion

This study of all patients tested for STIs at a large, urban, safety net hospital over an almost-three year period before and during the COVID-19 pandemic examined both temporal and location trends in STI testing and positivity rates. Overall testing and positivity rates were similar to national trends, with an early decrease in testing and increase in positivity rate, which then returned to near baseline in the LPP. The exception to this was chlamydia, for which rates remained higher than baseline throughout 2020, while nationally chlamydia diagnoses were decreased compared to the previous year. This study revealed that while the ED was already an important source of STI care before the pandemic, it played a larger role during the early pandemic, a trend that held true for subgroups of NHB, female, pregnant, and young patients, who were all more likely to use the ED for STI testing during the early pandemic. Furthermore, more than half of all positive tests originated in the ED, a proportion that also increased during the pandemic. The likelihood of a positive test from the ED increased during the EPP for all subgroups as well.

The pattern seen in this study of decreased testing and positivity rates very early on in the pandemic, followed by an increase in numbers of positive tests starting in June 2020, mirrors national trends that have been reported by the Centers for Disease Control and Prevention (10). Early pandemic increases in positivity rates may reflect lower overall testing numbers and greater barriers to seeking testing, with highly symptomatic patients more likely to seek testing. It is unknown exactly why the number of infections rebounded so strongly in May, reaching levels higher than those seen during the same periods in the years before and after. This may reflect a combination of factors, including lack of access to care during the early months of the pandemic, delayed care seeking due to fear of health care facilities, or increased transmission rates due to layoffs and changes in social behaviors related to the pandemic.

In this study the testing and positivity rates returned to near baseline by August 2020, while national reports showed a sustained elevation in gonorrhea diagnoses through the end of 2020. This may be attributable in part to the national shortage of test kits from September 2020 to January 2021, which affected the availability of testing at hospitals around the country, including UCM, at varying times. This may have led to an artificial deflation in the testing and positivity rates when in actuality the infection rates increased in parallel with nationally reported trends. Conversely, national chlamydia rates in 2020 were decreased compared to 2019, but this is thought likely not to represent a true decrease in infection rates, but rather changes in screening patterns nationally (10). As chlamydia and gonorrhea tests are conducted together at the study site, this may have helped to ameliorate any decreases that may have resulted from changes in testing patterns.

Although gonorrhea and chlamydia testing and positivity rates paralleled the trend seen among all STIs in this study, trichomonas deviated from this trend, with less temporal variation in testing rates and similar positivity rates throughout the study period. Over the entire study period, trichomonas testing was less common than gonorrhea or chlamydia. This may reflect a historical lack of clear recommendations about when to include trichomonas in STI testing, as well as the fact that gonorrhea and chlamydia testing are included together in one order, while trichomonas testing must be ordered separately. It is possible that clinicians tend to order trichomonas testing only for highly symptomatic patients or those returning for persistent symptoms after treatment, which would explain the lower variability in testing and positivity rates over the study period.

While it is generally understood that most STI testing originates in primary care clinics, STI clinics, or other outpatient care settings (5), few previous studies have examined the role of the emergency department in testing for and diagnosing STIs. Published reports have found 7% to 16% of patients seeking care for STIs utilized the ED (6, 7), although certain factors such as low income (6), male gender, young age, non-White race, and public insurance (7) were associated with greater likelihood of ED care seeking. Additionally, more recent data suggests that ED visits for STIs are increasing (12, 13). These demographic characteristics mirror those of the communities that often surround urban safety net hospitals. The current study, which took place at such a hospital, found that the ED was the origin of 20.9% of tests over the entire study period, which increased to 29.3% during the EPP, likely due to closures of outpatient clinics and a temporary move towards telehealth, leading patients with symptoms of an STI to seek alternative sources of care. This underscores the importance of the ED in the STI care continuum for vulnerable communities, especially during times of uncertainty or healthcare access limitations.

Even more strikingly, while only a fifth of STI tests originated in the ED, the ED was the source of approximately half of all positive tests. The fact that so many positive tests originated in the ED may suggest that patients who are symptomatic are more likely to seek care in the ED than other locations, which will be a potential area for further study. Preferential utilization of the ED by patients with STI symptoms could be related to actual or perceived difficulties or delays in accessing outpatient care, which were likely exacerbated by the COVID-19 pandemic. Many patients in vulnerable communities may be more likely to utilize the ED for all care (2) and less likely to have an established primary care doctor. With such a large proportion of positive STI testing originating in the ED, programs and partnerships are needed to ensure patient follow up for timely treatment, counseling, and HIV prevention education (14). A major barrier to ED STI testing is concern about informing patients of positive test results after discharge from the ED (5), as STI tests can take up to 48 h to return a result. In order to address this, the study site maintains a partnership between the ED and a Sexual Wellness Clinic (15) run by Infectious Disease physicians. This includes a team that assumes the burden of contacting patients with positive test results and offering STI treatment, preventive services, and sexual health education. Such a program may encourage more testing in the ED, lower barriers to STI testing, and in turn create an opportunity for education and linkage to ongoing outpatient care when the patient returns for treatment.

A subgroup analysis of pregnant women, considered a special population for STI screening by the CDC (16), revealed an even more substantial reliance on the ED for STI care. While the population of pregnant patients more often was NHB, younger, and relied on Medicaid/Medicare than the general population, this is thought to reflect the demographics of the community surrounding the medical center and the natural reproductive age range. Overall, 41.1% of all STI tests among pregnant women originated in the ED, which increased to more than half during the EPP. Women in the community surrounding the medical center have access to a variety of prenatal care options, from federally qualified health centers (FQHCs) with affordable and free options for care, to private obstetrics clinics. However, a growing body of research suggests that a large proportion of pregnant women may be visiting the ED during their pregnancies (17, 18), and recent evidence demonstrates significant racial and socioeconomic disparities in access to prenatal care (19). While the dataset is limited in its ability to identify all pregnant patients, and it is possible that this population made more use of outpatient clinics for STI testing than reported, the results of this study suggest that further investigation is needed to identify trends in STI care-seeking among pregnant women, as such a reliance on the ED would imply that pregnant women may favor the ED for their care or face significant barriers to accessing prenatal care.

Notably, the ED was already the source of 73.4% of positive tests among pregnant women before the pandemic, which increased to 82.1% during the EPP. Gonorrhea and chlamydia are frequently underrecognized and undertreated in pregnant women visiting the ED (20). Furthermore, previous studies have demonstrated that a only a small fraction of patients tested for gonorrhea or chlamydia in the ED are also tested for HIV and syphilis (21), and all pregnant women should be screened for HIV and syphilis to prevent devastating fetal complications (5). If pregnant women are preferentially seeking care for their STIs in the ED, then efforts should be made to ensure comprehensive screening takes place, including HIV and syphilis testing. These findings also suggest a great need for improved access to prenatal care, assistance navigating the medical system, and counseling and linkage to prenatal care programs in EDs.

### Limitations

Because this study was a retrospective review of EMR data, available data was restricted to that recorded in discrete fields in the EMR. This limited the ability to assess patient symptoms or the reason for STI testing, gender identity or sexual orientation, or socioeconomic indicators, such as education level or income. While socioeconomic status may have played a role in the increased reliance of certain populations on the ED during the pandemic, the overall trends are still relevant to the entire community and others like it around the country. Due to the same data limitations, certain pregnant patients may not have been identified as such, if no urine pregnancy testing was performed within one week of the STI testing encounter. This may have artificially decreased the number of women defined as pregnant in outpatient obstetrics and gynecology clinics, who may have already known they were pregnant or whose pregnancies were confirmed by means other than urine testing. Nonetheless, a large number of pregnant women did seek STI care in the ED in this study, which suggests the need for further research in this area and increased ED services for this population.

## Conclusion

This study examined testing and diagnosis trends for gonorrhea, chlamydia and trichomonas before and during the COVID-19 pandemic. Trends at this urban safety net hospital were similar to national trends in 2020, with a decrease in testing and positivity early in the pandemic but an early and sustained rise in positivity rates for gonorrhea and chlamydia beginning in May 2020. The ED proved to be an important source of STI testing and diagnosis among all populations, even more so during the early pandemic when access to outpatient services was limited. The significance of this reliance on the ED was most pronounced, however, among pregnant women, a large number of whom received their STI testing and diagnoses from the ED. This underscores the need for safety net hospitals to implement programs for linkage to prenatal care from the ED, support comprehensive STI testing for pregnant women in the ED, and address the barriers that pregnant women face in accessing routine prenatal care. Given the important role the ED plays in STI testing for all community members, consideration should be given to incorporating STI screening into routine ED care and devoting resources to education, prevention, and linkage to outpatient primary and sexual health care services. Such an intervention has the potential to reduce health care disparities by bringing comprehensive care and prevention services to the most vulnerable patients where they are most likely to seek care.

## Data Availability

The datasets presented in this article are not readily available because they contain sensitive patient information.
